# Target approach for prevention activities in dental service of closed government systems. Ukrainian concept

**DOI:** 10.1186/1878-5085-5-S1-A126

**Published:** 2014-02-11

**Authors:** Dmytro S Borkowski, Viktor V Zhuravel, Rostyslav V Bubnov

**Affiliations:** 1 Clinical hospital "Pheophania", Kyiv, Ukraine

## Scientific Objectives

Despite the progress of science, technology and health care dental morbidity is not only reduced, but some parameters are gradually increasing. Introduced in last century in Europe preventive measures significantly reduced the infestation of caries and its complications, especially in children. Unlike Europe in Ukraine dental services activities are focused mostly on the *treatment process* (i.e., secondary and tertiary prevention), especially on the treatment of caries and its complications. In many ways, this situation triggered very low levels of motivation as a patients so the medical and dental staff management. According to official statistics in 95.0% of patients visit their dentists for treatment, rather than for prophylaxis. Preventive part of the doctor in Ukraine (both private and public sectors) is rather recommendational than encouraging or motivational.

This preventive approach is due to the following problems:

- Lack of financial resources;

- No legal impact on physician administrative authorities in carrying out preventive measures;

- Unpreparedness no doctors or nurses (staff) to motivate the patient.

## Technological approaches

To a large extent, the problem has to be solved by prevention at the national level (focus on primary prevention) with obligatory participation of private clinics and offices, as well as special training and additional targeted funding area. Originally prevention must deal with the state, represented by the leaders through the first permanent medical programs, financially supported with a permanent monitoring system. We suggest evaluation prevention index as preventive coefficient calculated as the difference between the number of self-care patient visits per year and initial visits on treatment. To estimate the co-efficient propose the following scale:

- Excellent - from 4 to 2;

- Good - from 1 to 0;

- Satisfactory - from 1 to 3;

- Poor - from 4 and below.

For preventive factor and the possibility of calculating it is necessary to introduce a single dental document of patient (*dental passport*), where all visits to the dental office must be recorded.

## Results interpretation

Preliminary analysis shows that such an assessment may hold today, but only in **closed government systems** that have internal medical facilities. These institutions have the opportunity to implement and monitor all sections work through the administrative system in which there is a single valid documentation and ongoing monitoring of health during checkups. An important element of disease prevention - an opportunity doctor at the local level in the legal field to influence the government and eliminate caries inducing environmental factors.

## Conclusions

To implement such transformations in dental service and provide the population with quality dental care is necessary to develop and implement a comprehensive regional program that is designed for implementation in the medium term and will include, along with targeted use of budgetary funds, public hospitals and private dental structures.

## Expert recommendations

1. Prevention of dental diseases requires development and use of new medical technologies and organization.

2. Activity of dentist must be assessed from the perspective: the smaller the number of dental pathology identified in the attached population - the better its prevention works.

